# Determination of single molecule loading rate during mechanotransduction in cell adhesion

**DOI:** 10.1126/science.adk6921

**Published:** 2024-03-21

**Authors:** Myung Hyun Jo, Paul Meneses, Olivia Yang, Claudia C. Carcamo, Sushil Pangeni, Taekjip Ha

**Affiliations:** 1 Program in Cellular and Molecular Medicine, Boston Children’s Hospital, Boston, MA 02115, USA.; 2 Department of Pediatrics, Harvard Medical School, Boston, MA 02115, USA.; 3 Department of Biophysics and Biophysical Chemistry, Johns Hopkins University, Baltimore, MD 21205, USA.; 4 Department of Biophysics, Johns Hopkins University, Baltimore, MD 21205, USA.

## Abstract

Cells connect with their environment via surface receptors and use physical tension in receptor-ligand bonds for various cellular processes. Single-molecule techniques have revealed bond strength by measuring “rupture force” but it has long been recognized that rupture force is dependent on loading rate – how quickly force is ramped up. Thus, the physiological loading rate needs to be measured to reveal the mechanical strength of individual bonds in their functional context. Here, we developed an overstretching tension sensor (OTS) to allow more accurate force measurement in physiological conditions with single-molecular detection sensitivity even in mechanically active regions. We used serially connected OTSs to show that the physiological loading rate ranged from 0.5–4 pN/s and was about three times higher in leukocytes than in epithelial cells.

## One-Sentence Summary

Single-molecular tension sensors based on DNA overstretching reveal cellular force loading rate.

## Main Text:

Physical forces govern many cellular processes, including cell adhesion, migration, division, and differentiation (1–3). Single-molecule mechanical measurement technologies can reveal the strength of these bonds in the form of “rupture force” (4). The rupture force depends on the loading rate, which is a measure of how quickly the force is ramped up during such measurements (4–6). Loading rates in single-molecule measurements range from as low as ~0.1 pN/s in optical or magnetic tweezers up to ~10^6^ pN/s in atomic force microscopy (AFM), resulting in a substantial range of measured rupture forces even for the same bond, depending on the applied loading rate (7–9). Thus, the physiological loading rate must be determined to reveal the relevant mechanical strength of individual bonds in their functional context (10). However, the measurement of cellular force loading rate at the single-molecular level has remained a significant challenge.

To explore receptor-specific mechanotransduction by measuring the force exerted on individual receptors in living cells, molecular tension probes have been developed that transduce the detected force into fluorescence signals (11). Force-sensitive nanostructures, such as elastic peptides and DNA hairpins, have been employed to measure piconewton forces (3–20 pN) (12, 13). Meanwhile, force-rupturable structures, such as a DNA-protein complex and double-stranded DNA (dsDNA), have been adopted to limit the maximum tension exerted on a single receptor (4–56 pN) (14, 15). Such tension regulation studies have shown that a certain level of force on integrin is required for cell spreading, with the threshold differing significantly between integrin subtypes (10–40 pN) (14, 16). Thus, understanding diverse mechanotransduction processes demands accurate measurement and monitoring of force in the tens of piconewton range.

Here, we used DNA overstretching-induced dehybridization of an oligonucleotide to measure a broad range of single-molecular tension (16–95 pN) with minimal interference on the target receptors.

## Stretching force-induced oligonucleotide dehybridization

When stretched with a high force above 60 pN, long double-stranded DNA undergoes a structural transition from its native B-form to a partially single-stranded state (17), and short DNA can be fully dehybridized even at lower forces (18–20). We hypothesized that the dehybridization of oligonucleotides occurs at different levels of force depending on their sequence, both composition and length, allowing each sequence to serve as a digital tension sensor.

**Fig. 1.**
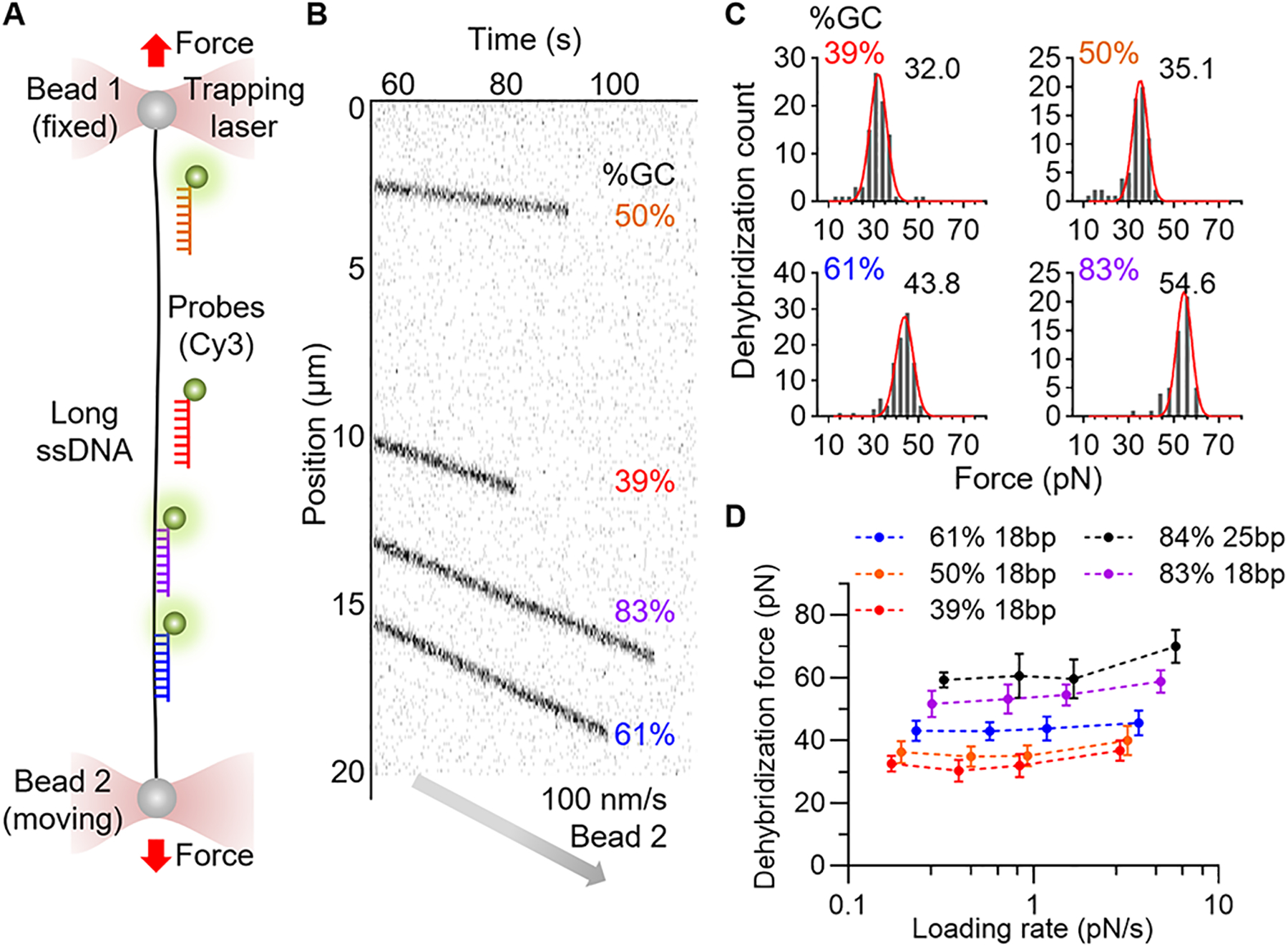
Stretching Force-Induced Oligonucleotide Dehybridization. Oligonucleotide dehybridization force was measured using optical tweezers. (**A**) Schematic of experimental setup. Fluorescently labeled oligonucleotides on a long ssDNA captured and stretched by optical tweezers. (**B**) Kymograph of four DNA oligonucleotides (18 bp, Cy3) on a ssDNA being stretched at a speed of 100 nm/s. The disappearance of fluorescent signals indicates dehybridization. (**C**) Distributions of dehybridization force (100 nm/s, RT). Gaussian fitting (red line) result is indicated. N = 89, 67, 96, and 52 obtained from seven or more different ssDNA. (**D**) Loading rate dependent dehybridization force. Dehybridization force of five DNA oligonucleotides was measured using four different stretching speeds (20, 50, 100, and 300 nm/s). The loading rates corresponding to the applied force were calculated using force-extension data and stretching speed (fig. S1). Data are mean±SD.

To test this, we sourced 18 base pair (bp) sequences with different GC-content (39%, 50%, 61%, and 83%) from the bacteriophage lambda DNA (λDNA) and fluorescently labeled the DNA oligonucleotides (Table S1). A double-stranded λDNA was captured in a microfluidic chamber using an optical tweezers system (fig. S1). The λDNA was biotinylated on both ends of the same strand and attached to streptavidin-coated polystyrene beads. A single-stranded λDNA (ssλDNA) template was generated by overstretching the λDNA with force exceeding 75 pN. The fluorescently labeled oligonucleotides were subsequently hybridized to the ssλDNA at low tension (~5 pN). Their positions were monitored using confocal scanning imaging and identified by their binding positions in λDNA (Table S1). We stretched the ssλDNA template at a constant speed of 100 nm/s. As the force applied to the ssλDNA increased, the probes began to dissociate (Fig. 1B). The dehybridization forces at room temperature (25 °C) of the four probes were 32 pN, 35 pN, 44 pN, and 55 pN, correlating with rising GC content (Fig. 1C).

Shorter probes (15 bp, GC 40% and 60%) dehybridized at an 11 pN lower force compared to 18 bp probes (fig. S1). A longer probe (25 bp, GC 84%) withstood force up to 60 pN, approaching the overstretching force of λDNA. To further expand the detection range, we evaluated a high GC content (18 bp, GC 83%) peptide nucleic acid (PNA) sequence. The dehybridization force rose from 55 pN for DNA homoduplex to 95 pN for the PNA-DNA hybrid (fig. S1), reflecting the higher stability of PNA-DNA hybrids (21).

To assess how loading rate influences dehybridization force, experiments were performed at stretching speeds of 20, 50, 100, and 300 nm/s, yielding loading rates from 0.2 to 5.9 pN/s for probes tested (Fig. 1D). As loading rate increased, dehybridization force generally increased, but the change remained modest across the tested range. Specifically, a probe (18 bp, GC 61%) exhibited dehybridization forces of 43.1±3.3 pN (SD; 0.23 pN/s), 43.0±2.9 pN (0.58 pN/s), 43.8±3.9 pN (1.2 pN/s), and 45.5±4.0 pN (3.7 pN/s) at the varying speeds. This modest increase suggests that dehybridization of these oligonucleotides accurately reports force near this range of loading rate. Moreover, by varying oligo sequence, length, and chemical composition, the detection force could be programmed.

Finally, we measured dehybridization forces at 37 °C, the temperature used for our live cell experiments. The force levels were lowered by 8–16 pN for the 18 bp probes and by 2 pN for the 25 bp probe (Fig. 1D).

## Overstretching Tension Sensor

**Fig. 2.**
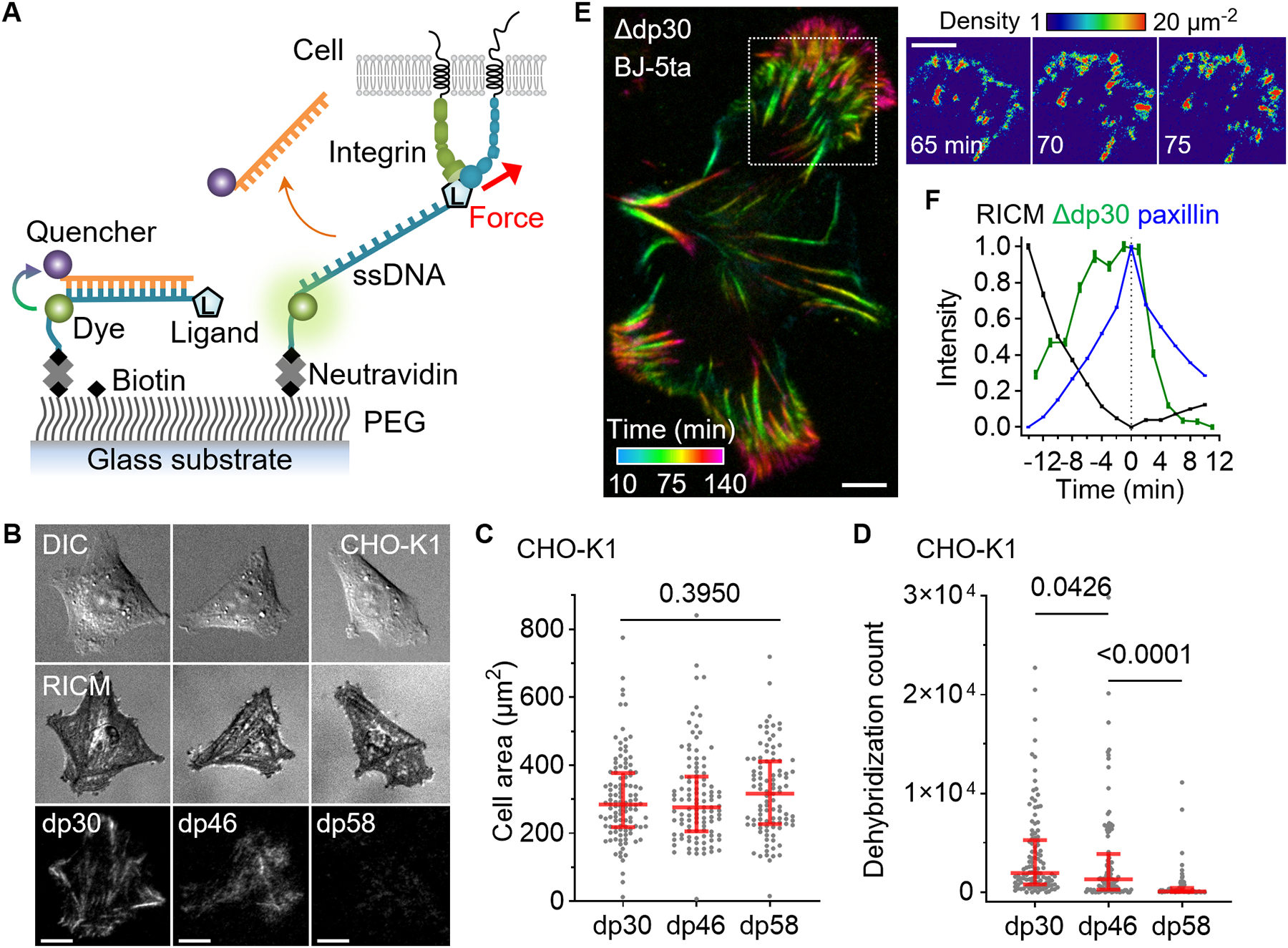
Overstretch tension sensor. Single-molecular force sensors were created based on force-induced oligonucleotide dehybridization. (**A**) Schematic of force detection using OTS. (**B**) Images of epithelial-like CHO-K1 cells seeded (2 hours) on densely immobilized (~1000 μm^−2^) dp30, dp46, and dp58 conjugated with a cRGDfK ligand. (**C**) CHO-K1 cell area on the three OTS coated surfaces. N = 112, 109, and 99 cells from three independent experiments. (**D**) The count of dehybridized OTS per cell. The red lines are median and interquartile range. P-values are from the Kruskal-Wallis test. (**E**) BJ-5ta fibroblast force-activated dp30 signal is temporally resolved at 5 min intervals. The density of force events quantified by measuring single Cy3 signals. (**F**) Temporal relation between RICM, dp30 signal increase, and paxillin-GFP signals. Pixel-wise signal traces were aligned to the peak of GFP signal and averaged (2830 pixels; mean±SE). BJ-5ta fibroblast spreading was monitored at 2 min time intervals. Scale bars, 10 μm.

Using the characterized sequences, we developed digital tension sensors (20), named Overstretching Tension Sensors (OTSs). First, we targeted αV integrins (16) by conjugating cyclo-RGDfK peptide (cRGDfK) to the 5’-end of ssDNA and biotinylating its 3’-end (Fig. 2A). To avoid steric hindrance, we incorporated a PEG linker to the peptide and appended six thymines at the 3’-end of DNA. A Cy3 fluorophore was attached to the 6^th^ thymine from the 3’-end (fig. S2). A complementary oligonucleotide, conjugated with a fluorescent quencher (BHQ2) at the 5’-end, was hybridized to form a DNA duplex. Consequently, the fluorescence signal is suppressed until the quencher strand is removed by stretching force.

Hamster epithelial cells (CHO-K1) were seeded onto a polyethylene glycol-coated glass surface functionalized with OTSs. OTSs, incorporating the DNA sequence with dehybridization forces of 30 pN, 46 pN, or 58 pN (37 °C, 100 nm/s), were denoted as dp30, dp46, or dp58. It is important to note that the dehybridization force is dependent on both temperature and loading rate as demonstrated. Thus, the results should be interpreted cautiously when these parameters may deviate from the calibrated conditions.

The cells spread well on all three surfaces, producing force-generated fluorescence signals in a streak pattern of mature focal adhesions (Fig. 2B). For comparison, cells were also seeded on surfaces coated with tension gauge tethers (TGT), DNA-based tension regulators that restrict the maximum integrin force to 33, 43, or 53 pN as predicted by physical modeling (14, 21). The cell area increased with higher allowed integrin forces on the TGT surfaces. In contrast, the cell area remained constant on the three OTS surfaces and was larger than on the TGT surfaces (Fig. 2C and fig. S3). Thus, OTS offers uniform and strong mechanical support regardless of its dehybridization force, unlike the rupturable TGTs that change cell adhesion responses depending on the tension thresholds. The dehybridization signal was less frequent for dp43 compared to dp30, and was very low for dp58, indicating that single integrin forces rarely exceed 58 pN (Fig. 2D). Similar patterns of spreading and force transmission were also observed in human foreskin fibroblasts, BJ-5ta (fig. S3).

OTS cumulatively records force transmission events, and the analysis of signal increase through time-lapse imaging allows for temporal resolution of force-generated signals (22). Human foreskin fibroblast spreading on the dp30 surface exhibited streaky force signal patterns, resulting from the accumulation of puncta at the growing tip of focal adhesions (Fig. 2E) (16, 21). Despite the high density of dp43 used to facilitate fibroblast spreading (16) (~1,500 μm^−2^), force signal calibration showed that OTS still achieved near single-molecule level detection sensitivity owing to its high quenching and annealing efficiency (Fig. 2E and Movie S1).

Focal adhesions are multiprotein structures that establish physical connections between internal actin bundles and the external substrate. The dp30 force signals showed a clear alignment with paxillin which served as a focal adhesion marker (fig. S4). To further investigate dynamic force transmission during focal adhesion formation and maturation, we analyzed the force signal time trajectories. The temporally aligned signal traces clearly showed that force transmission predominantly occurs during an increase in paxillin signal (Fig. 2F). During this phase, the Reflection Interference Contrast Microscopy (RICM) signal progressively decreased owing to close contact between the cell membrane and the substrate, signifying the strengthening of cell adhesion to the substrate. Following the peak of the paxillin signal, the force signal rapidly faded, suggesting that forces higher than 30 pN play an important role in focal adhesion maturation. In summary, simultaneous imaging of OTS, paxillin, and RICM shows that the OTS reports on single molecule force events during mechanical signaling.

## OTS Refreshing

**Fig. 3.**
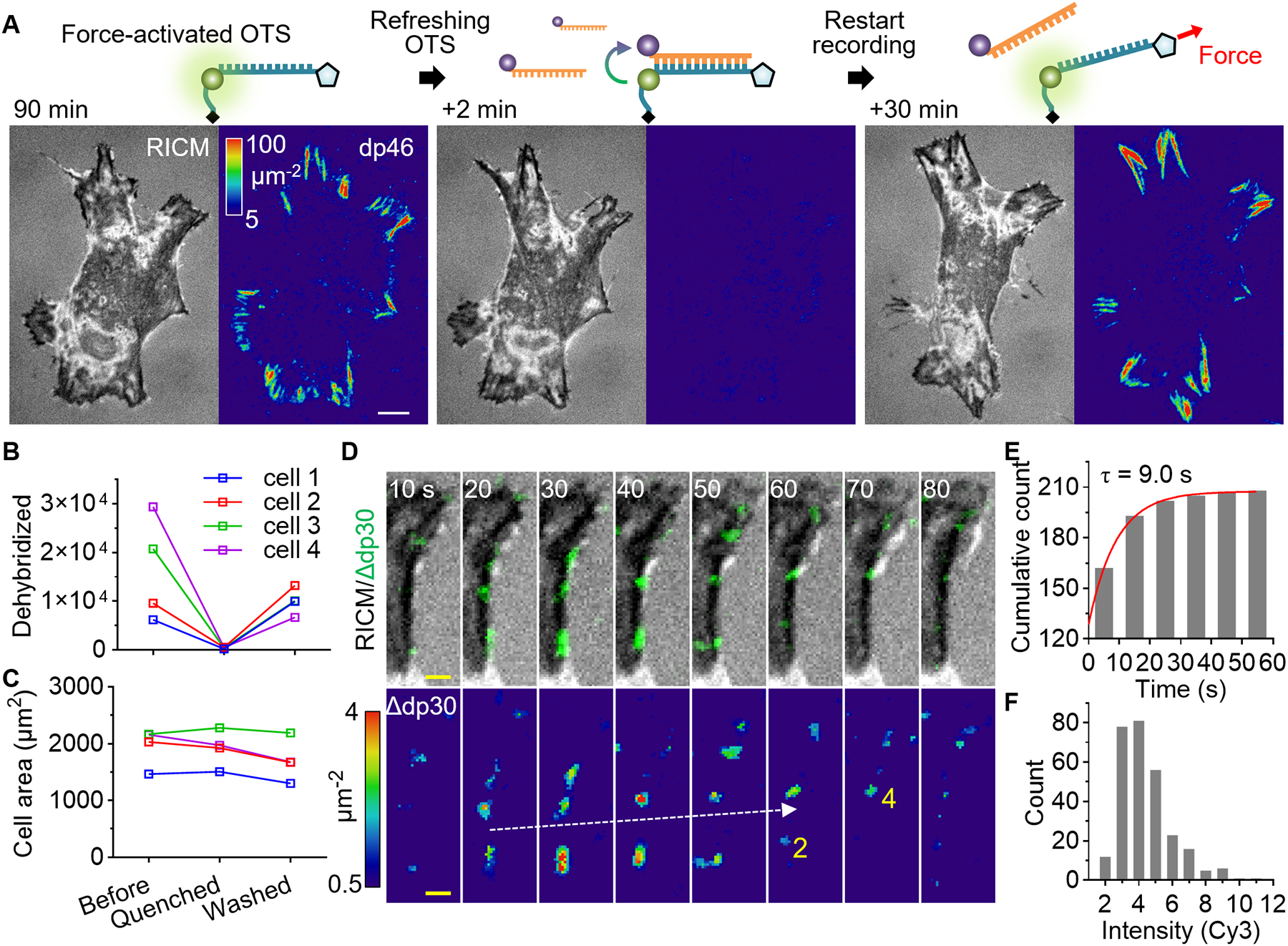
OTS refreshing. Refreshment of forced-activated OTS maintains the high detection sensitivity and reveals integrin subclusters within focal adhesions. (**A**) Quencher strands were injected (1 uM, 500 uL/min) into the imaging chamber to erase dp46 signals force-activated by fibroblasts. The force recording was resumed by washing out the quencher strands. (**B**) The total dp30 signal per cell in each step. N = 4 cells. (**C**) Cell area in each step. (**D**) Force detection within a mature focal adhesion on refreshed dp30 surface. The time resolved force signals (dp30 signal increase or Δdp30) are overlaid on the RICM images (top panel) and the intensity-calibrated images are shown after median filtering (bottom panel). The force transmission count for each frame is shown in yellow text for two spots. (**E**) The lifetime of force signal spots. Single-exponential fitting of the cumulative count was shown. (**F**) Fluorescence intensity histogram of individual spot normalized to single Cy3 signal. White scale bars, 10 μm. yellow scale bar, 2 μm.

Near focal adhesions, OTS force signals gradually accumulated over time owing to frequent force transmission, diminishing the sensitivity for detecting additional force transmission events. After 90 minutes of fibroblast spreading, we observed intense fluorescence signals concentrated in focal adhesions, corresponding to over 100 force-activated dp46 molecules per square micron (Fig. 3A). While 90% of the OTS remained intact, allowing for additional force detection without significant underestimation, the existing fluorescence signals reduced the detection sensitivity. To restore high detection sensitivity in these regions, we introduced quencher strands into the imaging chamber.

These strands hybridized to the ruptured OTS strands within two minutes, effectively erasing the force-generated fluorescence signals (Fig. 3B), and the subsequent removal of quencher strands allowed force recordings to resume. This approach of OTS refreshing maintained high detection sensitivity even after prolonged cell incubation, without disrupting cells (Fig. 3C).

To monitor force transmission within matured focal adhesions, we imaged dp30 in fibroblasts at 10 s intervals. Intriguingly, we observed submicron-sized spots that slowly translocated along the focal adhesion (Fig. 3D). The lifetime of spots was 9.0 s (Fig. 3E), and only a few force transmission events were observed per 10 s (Fig. 3F). These transient small clusters were also detected with dp46, but less frequently (fig. S5). Overall, OTS refreshing revealed integrin subclusters within integrin-dense focal adhesions, which may collectively share the mechanical load and mediate dynamic and localized force transmission.

## Integrin force loading rate measurements

The mechanical stability of biochemical bonds between molecular components—including ligands, surface receptors, adapter proteins, and actin filaments—is dependent on the force loading rate. Single-molecule force measurements have revealed the rupture force of bonds or unfolding force of proteins (7–9). However, the specific loading rate that is most pertinent in vivo for each scenario remains to be clarified. Furthermore, consideration of the loading rate is fundamental to the force measurement itself because the structural change of a tension probe depends not only on the force level but also on the loading rate (23).

**Fig. 4.**
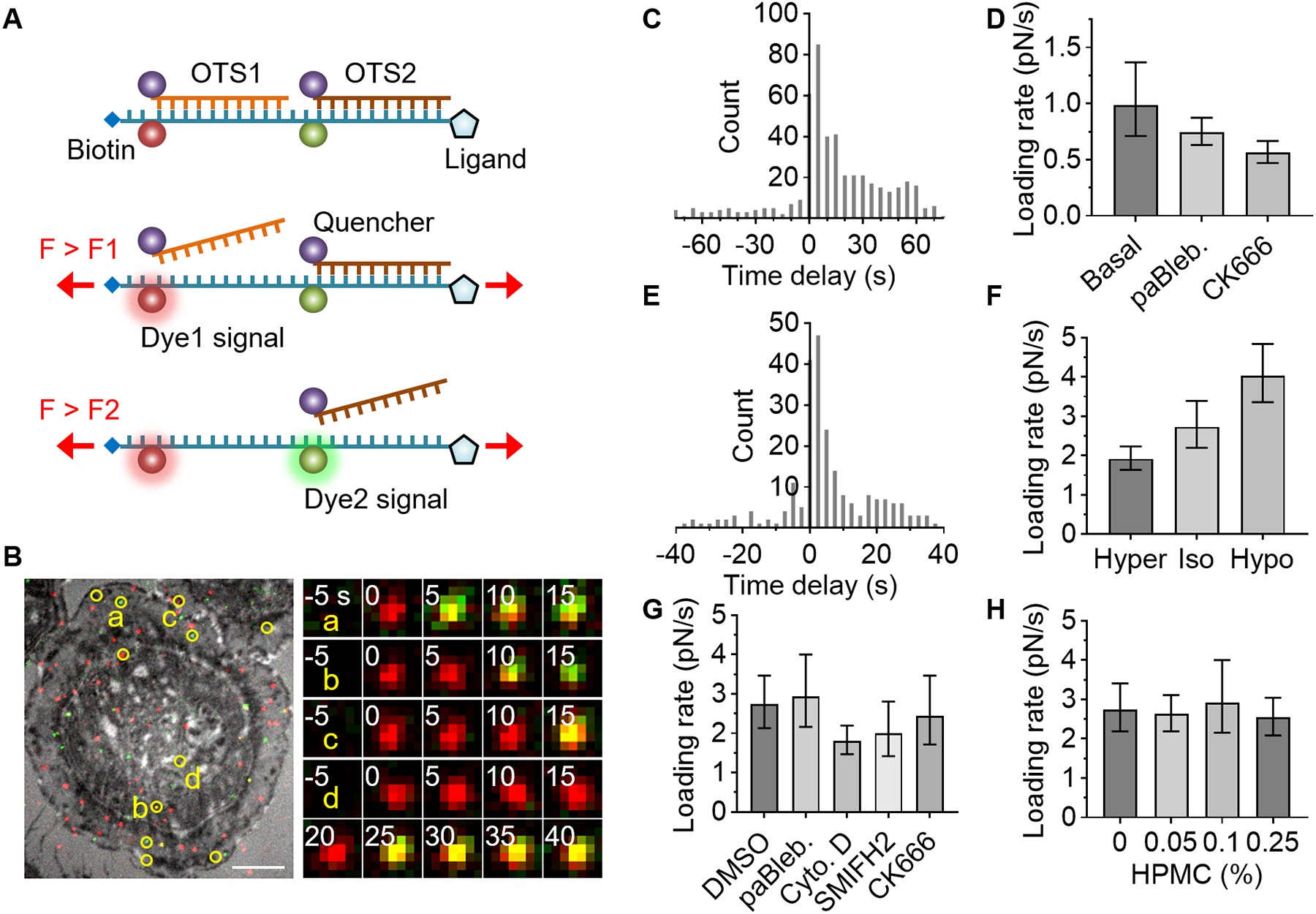
Cellular force loading rate measurement using serially connected OTS. A serially connected OTS detects two levels of force, enabling loading rate measurement. (**A**) Schematic of two-level force detection using OTS. (**B-D**) RGD-binding integrin force in epithelial cells (U2-OS). The ligand used is cRGDfK. (**B**) Two OTS signals (red for dp16-Atto647N and green for dp30-Cy3) are overlaid on the ventral surface image (RICM). Colocalized signals were marked in yellow circles (Atto647N and Cy3). Time-lapse images of the spots are shown. Scale bars, 10 μm. (**C**) Histogram of time delay between dp16 and dp30 signals (5 s interval). (**D**) Effect of cytoskeletal inhibitors on loading rate in U2-OS cells: para-amino-Blebbistatin (10 μM) or CK666 (50 μM). (**E-H**) Integrin α4β1 force in monocyte (THP-1) was measured using LDVP. (**E**) Histogram of measured time delay in basal condition (2.5 s interval). (**F**) Loading rate in a hypertonic, isotonic, or hypotonic medium. (**G**) Loading rate with DMSO (0.02%) or a cytoskeletal inhibitor: para-amino-Blebbistatin (10 μM), Cytochalasin D (0.5 μM), SMIFH2 (1 μM), or CK666 (10 μM). (**H**) Viscosity effect on loading rate. Hydroxypropyl methylcellulose was added. Data is combined from three independent experiments. Data and error bars indicate the result of single-exponential fitting and 90% confidence intervals (**D, F-H**). See fig. S7 for additional information.

At least two levels of force should be detected within a single tension sensor to measure loading rate. To achieve this, we labeled dp16 with Atto647N and dp30 with Cy3, and serially connected them (Fig. 4A). In dp16/dp30, the Atto647N signal (red) appeared when the applied tension was higher than 16 pN and Cy3 signal (green) appeared when the force exceeded 30 pN. To monitor single-molecule force signals without overlap, we immobilized a low density (0.7 μm^−2^) of dp16/dp30 (fig. S6). The density of fluorescent spots was even lower because most OTSs were in the quenched state. To facilitate cell spreading, integrin ligands were additionally immobilized through a ssDNA linker of equal length. A density of 110 μm^−2^ of cRGDfK ligands was sufficient to support epithelial cell (U2-OS) spreading (Fig. 4B).

After cell spreading and subsequent OTS refreshing, we observed the emergence of dp16 signal (red), followed by dp30 signal (green) on the same diffraction-limited spot, indicating a 13.2 pN increase in force across a single integrin-ligand bond (Fig. 4C, Movie S2, and fig S1). Such colocalized spots were observed only in the cell-occupied area (Movie S2 and Movie S3). The histogram of the delay time between dp16 and dp30 signals exhibited a single exponential decay, with a decay time of 13.4 s (fig. S9), corresponding to an average loading rate of 0.99 pN/s (ΔF/Δt = 13.2 pN/13.4 s). This observed loading rate aligned closely with the loading rate used in the force calibrations (0.4–0.9 pN/s for 16–30 pN at 37 °C), demonstrating consistency between the cellular force and OTS calibration loading rates (fig. S1). The probability that two different integrins sequentially bind and activate the same OTS is expected to be very low because only 0.6% of sensors are fluorescently labeled and less than 0.1% of the labeled sensors are activated per time point under our measurement condition (0.2 s exposure every 5 s).

Actomyosin activity and actin polymerization drive cellular forces. We explored their roles in force transmission and loading rates using cytoskeletal inhibitors on epithelial cells. Para-amino-Blebbistatin (10 μM), inhibiting actomyosin activity, did not alter cell spreading on OTS (dp16, dp30, and dp46) but reduced force transmission events by 30% (fig. S8) and decreased the loading rate to 0.74 pN/s (Fig. 4C). CK666 suppresses actin branching by inactivating Arp2/3 (24). Similar to BJ-5ta fibroblast (16), CK666 (50 μM) did not affect epithelial cell spreading area or force transmission frequency (fig. S8). Notably, however, CK666 lowered the loading rate to 0.56 pN/s (Fig. 4C), highlighting actin branching’s key role in force development. Cytochalasin D (10 μM and 0.5 μM), a potent inhibitor of actin polymerization, abolished cell spreading and force transmission (fig. S8), precluding loading rate measurement.

Leukocytes migrate from the bloodstream to inflammation sites via sequential adhesive interactions. The force loading rate on integrin α4β1 in leukocytes was measured using dp16/dp30 conjugated with an α4β1-specific ligand, LDVP peptide (MUPA-LDVPAAK) (16). Monocytes (THP-1) adhered to the LDVP-coated surface (0.7 μm^−2^ dp16/dp30, 11 μm^−2^ in total), transmitting high forces (Movie S3). The average loading rate between 16 pN and 30 pN was 2.7 pN/s (Fig. 4E and fig. S7), about three times higher than that of epithelial cells.

Given that actin retrograde flow relies on the cell membrane’s reaction force, the physical properties of the membrane likely play a crucial role in transmitting force to the substrate. In hypertonic medium, which reduces the osmotic pressure, a lower loading rate was observed (1.9 pN/s) (Fig. 4F). Conversely, hypotonic conditions yielded a higher loading rate (4.0 pN/s). Thus, membrane tension may influence the loading rate, possibly by facilitating actin retrograde flow.

Cytochalasin D, at 10 μM, inhibited monocyte spreading and force transmission. However, at 0.5 μM, cell area was not significantly reduced and force transmission persisted (fig. S9), allowing loading rate measurement. The loading rate (1.8 pN/s) was significantly lower than that of the control (2.8 pN/s; Fig. 4G), underscoring actin polymerization’s importance in force development.

Other actin polymerization inhibitors, SMIFH2 (1 μM) and CK666 (10 μM), also reduced the loading rate, but the effects were less pronounced. Notably, inhibition of actomyosin activity did not significantly decrease monocyte spreading area, force transmission frequency (dp30), or loading rate, implying that actin polymerization primarily drives monocyte adhesion (Fig 4G and fig. S9).

The viscosity of blood (3.5–5.5 cP) is higher than that of the cell medium, which typically measures around 1 cP at 37°C. We introduced hydroxypropyl methylcellulose, increasing medium viscosity, up to 0.25%, thereby elevating the viscosity to 9 cP (25). The loading rate was not significantly affected by the viscosity in this range (Fig. 4H).

Collectively, we conclude that the single-molecular force loading rate through integrins is around a single pN/s. The loading rate in adhering monocytes was higher than in epithelial cells, suggesting their capability to swiftly assess their mechanical environment during the dynamic phases of leukocyte adhesion and extravasation. We speculate that a similar range of loading rates may apply to many other mechanosensitive receptors, including cadherin, T-cell receptor, B-cell receptor, and Notch receptor because these receptors are also closely associated with actin filaments (26–29). Notably, cadherins, which primarily mediate cell-cell adhesion, share adaptor proteins with integrins (27).

## Discussion

Molecular tension probes offer promise in revealing receptor-specific mechanotransduction (30) but they had limitations including their narrow force measurement range, accuracy, and calibration without regard to temperature or loading rate. Our work demonstrates that stretching-induced oligonucleotide dehybridization is particularly suited for developing tension sensors for a wide range of force level.

Dehybridization increases the sensor’s length by only 3 nm for 18 bp DNA (19), and likely as a result, cells exhibited similar spreading across all tested OTSs, suggesting that dehybridization does not interfere with mechanosensing through integrins. The OTS can record transient or infrequent force transmission events without continuous illumination of living cells, preventing phototoxicity. Its refreshable nature maintains sensitivity in receptor-dense and mechanically dynamic regions like focal adhesions, extending the observation time beyond early adhesion or short-term imaging (22).

Moreover, configuring an OTS in a serial connection opens avenues for measuring cellular loading rates, a pivotal parameter in cellular mechanics including rigidity sensing (31–34), across a wide range of force. Notably, force ramping events have been captured only for T cell receptors within a low-force range (<4 pN) (35). Previous studies have shown that a 10 pN integrin tension difference markedly alters cell spreading (14, 16). Given the loading rates of 0.5–4 pN/s determined in our study, cells would have only a few seconds to sense the mechanical environment through single integrin molecules.

We measured the loading rate for ligands tethered to rigid glass via a PEG linker. On softer surfaces, the effective loading rate might be lower as ligands tend to move in the direction of the applied force. The resulting tilt in balance toward receptor-ligand unbinding before mechanical activation of integrin signaling is likely to be the fundamental mechanism underlying rigidity sensing (33).

Exploring loading rates is expected to uncover cell mechanics not observable through force level measurements alone. The understanding derived from integrin force loading rates can also be extended to the mechanics of cytoplasmic adaptor proteins. Notably, talin and filamin undergo substantial structural changes to relay force from membrane receptors to the cytoskeleton (36). Magnetic tweezers studies indicate their unfolding force varies with loading rates, from 0.1 to 10 pN/s, a range deemed physiologically pertinent (37–39). These findings, consistent with our measured loading rates, imply that adaptor proteins could regulate cell adhesion by detecting integrin force loading rates.

## Supplementary Material

Movie S1

Movie S2

Movie S3

Supporting Online Materials

## Data Availability

All data are available in the main text or the supplementary materials. This study used stabilized cell lines (U2-OS paxillin-GFP and BJ-5ta paxillin-GFP), and the cell lines are available upon reasonable request. Python scripts for optical tweezers experiments and MATLAB scripts for two-level OTS analysis are deposited to Zenodo (10.5281/zenodo.10635585) and also available on GitHub (https://github.com/1molecule/OTS).
