# Supplementary material for: Determination of single molecule loading rate during mechanotransduction in cell adhesion
Source: Science. Author manuscript; Available in PMC 2024 Mar 28. (PMC10977658; doi:10.1126/science.adk6921)
Supplement: Supporting Online Materials [file NIHMS1973891-supplement-Supporting_Online_Materials.pdf]

Science
AAAS

Supplementary Materials for

Determination of single molecule loading rate during mechanotransduction

Myung Hyun Jo, Paul Meneses, Olivia Yang, Claudia C. Carcamo, Sushil Pangeni and Taekjip Ha

Corresponding author: taekjip.ha@childrens.harvard.edu

The PDF file includes:

Materials and Methods
Figs. S1 to S9
Tables S1

Other Supplementary Materials for this manuscript include the following:

Movies S1 to S3

Materials and Methods

Measurement of oligonucleotide dehybridization force

The dissociation of fluorescently labeled oligonucleotides from ssDNA was monitored using an optical tweezers system equipped with microfluidics and confocal fluorescence imaging (LUMICKS, C-Trap® Optical Tweezers). The continuous-wave optical tweezers were operated in a dual-trap regime. Five laminar flow channels were maintained, each containing streptavidin-coated beads, biotinylated λDNA, PBS, imaging buffer, and oligonucleotides, respectively, at room temperature. Two streptavidin-coated polystyrene beads (4.34 µm; Spherotech, SVP-40-5) were trapped, calibrated, and moved to capture λDNA. The captured λDNA was then stretched until one strand was completely peeled off. The states of dsDNA and ssDNA were confirmed by examining force-extension curves each time. A wormlike chain model was used to check dsDNA (contour length of 16.49 µm for 48.5 kb, persistence length of 46 nm, and stretch modulus of 1000 pN). Freely jointed chain model was used for ssDNA (contour length of 27.16 µm, persistence length of 0.9 nm, and stretch modulus of 900 pN). The ssDNA template was incubated in the oligonucleotide probe channel for a few seconds and then transferred to the imaging buffer channel (a glucose oxidase oxygen scavenging system, NaCl 150mM, Tris-HCl 20mM, pH 7.5).

The ssDNA template was stretched by moving only one bead at a constant speed, and confocal scanning (532 nm laser; 100 nm pixel size, 500 ns pixel time) was used to monitor oligonucleotide probe location and generate a kymograph depicting the stretching procedure. This procedure was automated using a python script for Bluelake software (LUMICKS). The time point of probe disappearance was determined based on the kymograph and the corresponding force applied to the ssDNA template was recorded using a python script. During stretching at a constant speed, the loading rate steadily increases over time. Loading rates, corresponding to force levels, were estimated by multiplying the change in force per distance by the used stretching speed. The change was derived from a representative force-extension curve of a ssDNA template. The obtained loading rate increased almost linearly with the applied force in the observed region, thus linear regression was employed to interpolate the data.

DNA and oligonucleotide preparation for optical tweezers experiments

To make a long ssDNA by stretching, only one strand of λDNA (Roche, 10745782001) was biotinylated at both ends using the protocol previously established by Candelli et al (*40*). A linearized λDNA (NEB, M0201S) has cohesive overhangs (12 nucleotides) at its two 5' ends. Three oligos were used to biotinylate both ends: oligo 1 (5'- GGG CGG CGA CCT GGA CAA -3'), oligo 2 (5'- AGG TCG CCG CCC TTT TTT TT*T T*TT* -3'), and oligo 3 (5'- T*TT* TT*T TTT TTT AGA GTA CTG TAC GAT CTA GCA TCA ATC TTG TCC -3'). T* indicates biotinylated thymine. T4 polynucleotide kinase (NEB, M0201S) was used to phosphorylate the 5' terminus of λDNA, oligos 1, and oligo 2. Oligo 1 was annealed to one of the λDNA 5' overhang so that the λDNA has one 3' overhang and one 5' overhang. Oligo 3 was annealed to the 3' overhang and the construct was ligated using T4 DNA ligase (NEB, M0202S). Next, oligo 2 was annealed to the remaining 5' overhang of λDNA and ligated. The ligated construct was purified by dialyzing

in storage buffer (10 mM Tris-HCl pH 8, 1 mM EDTA, 50 mM NaCl) and stored at 4 °C for up to one month. This biotinylated λDNA was converted to a single-stranded DNA template by overstretching in optical tweezers system before dehybridization force measurements.

To prepare fluorescently labeled oligonucleotides (dehybridization probes), Cy3 labeled (5' end) DNA was obtained (IDT, custom DNA) or N terminal of PNA (PNA Bio, custom PNA) was labeled using Cy3 NHS ester (Cytiva, PA13101). The probes were purified using reverse-phase HPLC after labeling.

Overstretching Tension Sensors

For the overstretching strand of OTS, oligonucleotides were decorated with a peptide ligand (5' end), a fluorescent dye (internal), and a biotin (3' end). The procedure and structure of OTS are depicted in fig. S2. DNA oligonucleotides with three modifications (thiol at 5' end, internal amino modified thymine, and biotin at 3' end) were synthesized (IDT, custom DNA). Six thymine was added to the 3' end of the characterized sequences as a flexible linker to minimize steric hindrance. The first thymine was also used for dye labeling. Cy3 NHS ester was reacted to the internal amine. Peptide ligands were conjugated to the 5' end of DNA. For αV integrins, cRGDfK with a spacer (Vivitide, PCI-3696-PI) was conjugated using sulfo-SMCC (Thermo Fisher Scientific, 22622). The terminal free amine on the cRGDfK reacted with the NHS ester group of sulfo-SMCC, and the maleimide group of sulfo-SMCC was subsequently conjugated to the thiol at the 5' end of DNA. For integrin α4β1, MUPA-LDVPAAK (Tocris, 7020) was conjugated using copper-free strain-promoted azide–alkyne cycloaddition (SPAAC). The lysine of the LDVP peptide was reacted with Azide-NHS (Lumiprobe, 53720) in DMSO with triethylamine (1%), and the thiol of DNA was reacted with DBCO-PEG4-Maleimide (Click Chemistry Tools, A108P) in HEPES buffer (pH 7.3). The products with Azide and DBCO were then reacted in HEPES buffer (pH 7.3). Each product was purified using reverse-phase HPLC or ethanol precipitation.

For the two-level OTS, a biotinylated part and a ligand-conjugated part were separately fluorescently labeled or liganded before DNA ligation. The ligand part also contained six thymines but lacked biotin at its 3' end. The ligand was conjugated as described above. The 5' end of the biotinylated part was phosphorylated for ligation. The two parts were annealed to a DNA splint containing complementary sequences for both parts, and the nick was ligated using T4 DNA ligase (NEB, M0202S). The product was denatured in NaOH and purified using reverse-phase HPLC.

For quencher strands, BHQ-2 was conjugated to the 5' end of the DNA oligonucleotide (Biosearch Technologies, custom DNA). For the quenched OTS, two-fold quencher strands were added to the overstretching strands (1 μM) to ensure complete annealing and incubated for 10 min in TE150 buffer (pH 8.0; Tris-HCl 10mM, EDTA 0.1mM, NaCl 150mM).

Cell culture and transfection

Three types of human cell lines (fibroblast, epithelial-like cell, and monocyte) were investigated in this study, along with one mouse epithelial cell line for comparison with TGT assay which has been extensively tested with the mouse epithelial cell line. Cells were maintained at 37 °C under 5% carbon dioxide, with passage performed every three days. CHO-K1 (ATCC, CCL-61) was

cultured in Ham's F-12K Medium (Gibco, 21127022). BJ-5ta (ATCC, CRL-40011) and U2-OS (ATCC, HTB-96) were cultured in high glucose DMEM (Sigma, D5796). THP-1 (ATCC, TIB-202) was cultured in RPMI 1640 (Gibco, 11875093). The medium was supplemented with 10% heat-inactivated fetal bovine serum (Corning, 35-011-CV) and 1X Antibiotic-Antimycotic solution (Gibco, 15240062). Cell lines expressing Paxillin-TagGFP2 were generated using lentiviral transduction (Sigma-Aldrich, 17-10154). Following transduction, flow cytometry was employed to sort the cell population based on the GFP signal.

Cell adhesion experiments

For adherent cells, the cell dish was rinsed with PBS and filled with 0.05% Trypsin-EDTA (Gibco, 25300062). Following a 2 min incubation at 37 °C, the dish surface was gently rinsed by pipetting. The cell solution was then combined with a complete cell culture medium containing fetal bovine serum to neutralize trypsin. This step was skipped for suspension cells. Subsequently, the cells were spun down and resuspended with an imaging medium, Leibovitz's L-15 Medium (Gibco, 21083027) supplemented with 2 g/L glucose, then seeded on a tension sensor-coated surface at an approximate density of 30 cells/mm^2.

The imaging culture medium (L15) was diluted by a factor of two while maintaining the concentrations of glucose, calcium ions, and magnesium ions, to prepare a hypotonic medium. The hypertonic medium was prepared by adding 200 mM sucrose to the isotonic imaging buffer. Cells were preincubated in the solution for 10 min before seeding. For cytoskeletal component inhibition, cells were incubated for 5 min in the presence of para-amino-blebbistatin (Axel, ax494682), Cytochalasin D (Sigma-Aldrich, C8273), CK666 (Sigma-Aldrich, SML0006), or SMIFH2 (Sigma-Aldrich, S4826) before seeding. For viscosity control, a surface-treated hydroxypropyl methylcellulose (DuPont, METHOCEL J75MS) was added to the imaging medium, and cells were preincubated in the solution for 10 min before seeding.

Live cell imaging

The imaging area was humidified, and the temperature was maintained at 37 °C. A Nikon Eclipse Ti microscope equipped with a solid-state white light source (Lumencor, SOLA SE II 365), Nikon perfect focus system, custom total internal reflection fluorescence microscopy (TIRFM) module, and custom reflection interference contrast microscopy (RICM) module was controlled by Nikon NIS-Elements. Nikon 10X objective (CFI60 Plan Fluor) and 60X objective (CFI60 Apochromat TIRF) with DIC filter sets were used. A custom filter (Chroma, zet488/543/638/750m) and lasers (Coherent, 488 and 641 nm; Shanghai Dream Lasers Technology, 543 nm) were used for TIRFM. The long-pass filter (Thorlabs, FGL645) and the custom filter (Chroma, zet488/543/638/750m) were used with the Xenon lamp for RICM. Images were recorded using an electron-multiplying charge-coupled device (EMCCD; Andor iXon 888).

Surface passivation and functionalization

Cover glasses (No 1.5; VWR, 16004-312) were densely PEGylated to minimize non-specific interactions such as the binding of cell-secreted adhesion proteins. Coverslips were cleaned by

piranha etching solution (a 3:1 mixture of sulfuric acid and hydrogen peroxide) for 20 min and rinsed with water thoroughly. The coverslips were incubated in methanol with 3% APTES (Sigma-Aldrich, 440140) and 5% acetic acid for 30 min followed by water rinsing and drying. Two coverslips were sandwiched with polyethylene glycol solution (75 ul, ~100 mg/ml; 1:19 mixture Biotin-PEG-SVA-5000 and MPEG-SVA-5000; Laysan Bio) for 4 hours at room temperature. The surfaces were washed thoroughly and dried using nitrogen gas after each step. A silicon gasket (Grace Bio Labs, 103280) was attached to a coverslip to form multiple dishes. For buffer change experiments, microfluidic channels were crafted by sandwiching a coverslip and a clean quartz slide with holes for tubing or pipette tip connection. Double-sided tape was used as a spacer (Scotch, 665). Neutravidin (Thermo Fisher Scientific, 31000; 0.4 mg/ml) was added to the dishes or channels for 10 min and washed out with PBS. Tension sensors were added to the dishes or channels and washed. For regular cell adhesion experiments, 3 uL of 100 nM OTS solution was placed on the neutravidin-coated surface in a dish for 10 min in a humid condition. For two-level OTS experiments, 250 pM OTS solution was incubated in the microfluidic channel for 5 min and additional ligands (50 nM for cRGDfK and 5 nM for LDVP) were incubated for 5 min, followed by washing with imaging medium.

Cellular force loading rate measurement

For epithelial cells, cRGDfK-dp16(Atto647N)/dp30(Cy3) was immobilized at a low density (~0.7 μm-2) for single-molecule monitoring. Additionally, cRGDfK ligands were immobilized at a higher density (~110 μm^{-2}) via a ssDNA linker of equal length (GGT GAT GCG GAG AGA TGG TTT TTT CGG GGC CGT GGC AGT CGC TTT TTT) to support cell spreading. The ssDNA linker included a thiol modification at the 5'-end for ligand conjugation and a biotin at the 3'-end for immobilization. For leukocytes, LDVP-dp16(Atto647N)/dp30(Cy3) was immobilized (~0.7 μm^{-2}) and LDVP ligands were additionally (~11 μm^{-2}) immobilized through the same ssDNA linker. To remove the fluorescence spots generated during epithelial cell spreading, OTS refreshing was conducted before the loading rate measurement. This refreshing step was not required for leukocyte adhesion. The time delay between two OTS signals was analyzed as described separately. The loading rate calculated from the time delay represents the average loading rate between two force levels detected by dp16 and dp30. However, due to the non-linear nature of integrin force increase, the instantaneous loading rate may vary. Furthermore, the loading rate may differ across different force regions and different cellular locations/contexts.

Image analysis

Image analysis was performed using ImageJ (v1.53) and MATLAB (R2023a). The dark regions in RICM images, representing areas of close cell-surface adhesion (much smaller than 300 nm), were analyzed based on segmentation using MATLAB. Lateral drift correction was achieved by spot tracking through the ImageJ TrackMate plugin (*41*) and MATLAB. Time-resolved OTS signal analysis involved collecting pixel-wise intensity changes, which were then median-filtered over time (N=3) to mitigate defocusing-induced noise.

The analysis of two-force-level OTS data followed these steps: Time-lapse images of RICM, GFP (paxillin-GFP cells), Cy3, and Atto647N were obtained. Lateral drift correction was performed by tracking Atto647N single-molecule spots. Correction for chromatic error was done by comparing fluorescent bead images (Thermo Fisher Scientific, F8831) in each color channel using MATLAB. Single-molecule spots (Cy3 and Atto647N on OTS) were detected and tracked using ImageJ, with analysis restricted to immobile spots. Fluorescent spots exhibiting both fluorophore signals were collected, and time delays were analyzed using MATLAB. Overlapping or blinking spots were filtered out based on their location. Seamless analysis was facilitated by running ImageJ within MATLAB using the ImageJ-MATLAB extension (*42*).

Molecular density quantification

To determine the density of OTS and force-activated OTS in a dense condition, we measured the intensity of single fluorophores by analyzing the time traces of their fluorescent signals as depicted in fig S6. The reduction in intensity caused by dye photobleaching was collected and the median value was used to calculate the dye density. Calibration imaging was conducted under conditions equivalent to the corresponding experiments, with the exception of exposure time. For low-density conditions with minimal overlapping, OTS was immobilized using varying concentrations, and the number of spots was directly counted. The data was used to extrapolate the density for intermediate conditions.

Tension Guage Tethers

Tension gauge tethers (TGTs) with 18 bp DNA duplex were used (*21*). The sequence of the duplex region is GGC CCG CAG CGA CCA CCC (ligand conjugated strand). The nominal values of TGT tension tolerance were used as estimated by previous reports (*14*). The tension tolerance of each TGT was not experimentally measured and the value depends on loading rate which is a measure of how quickly applied force increases.

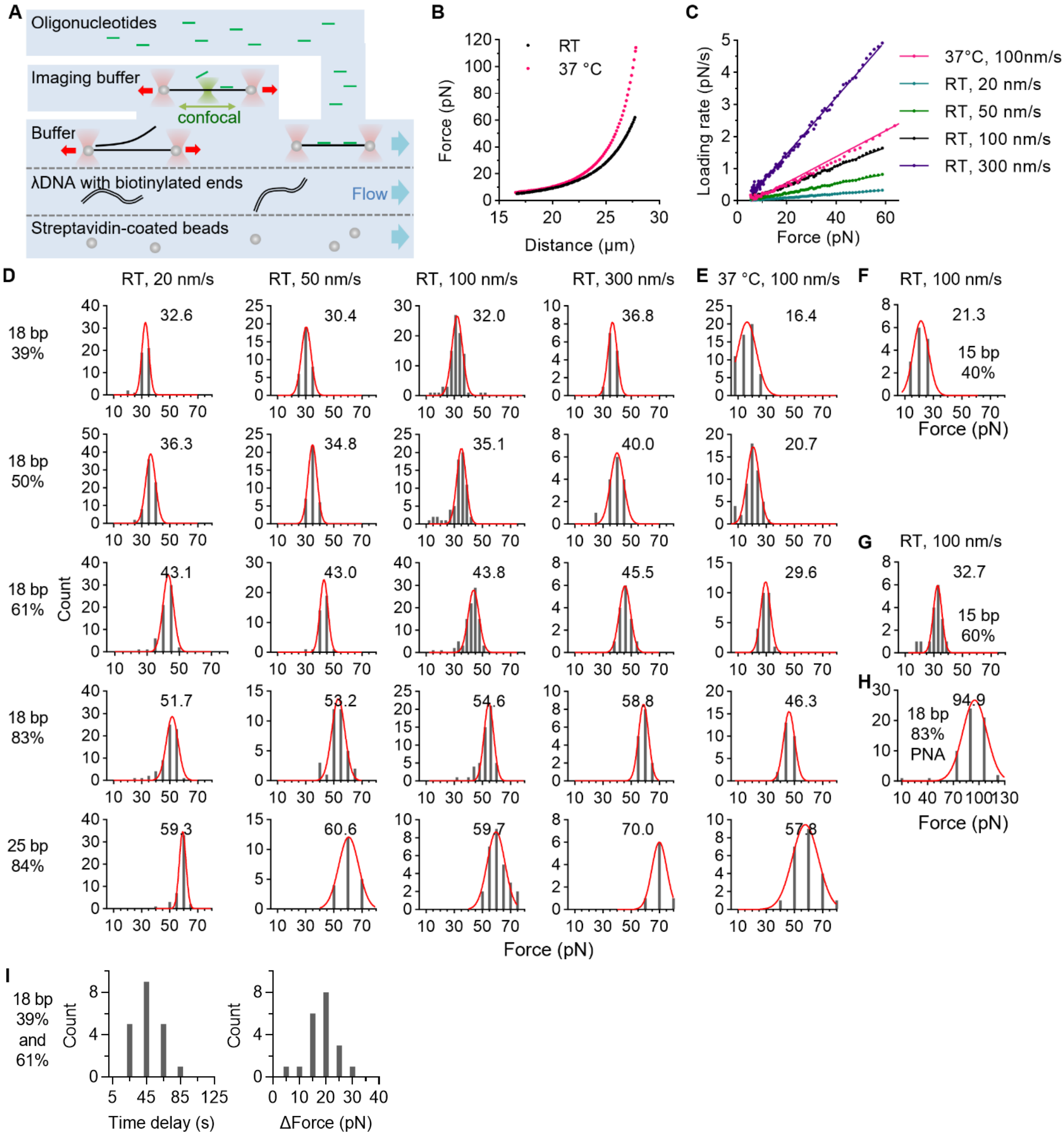

Fig. S1. Dehybridization force measurements

(A) Schematic of optical tweezers experiments. Laminar flow channels were maintained using an air pump. Two streptavidin-coated beads were trapped and moved to the DNA channel to capture a DNA molecule. The captured DNA was then stretched to create a ssDNA, confirmed by analyzing extension curves. The ssDNA was moved to the probe channel. Subsequently, the probe hybridized DNA was moved to an imaging area and stretched at a specified speed. The probe dehybridization was monitored using confocal scanning.

(B) Representative force-extension curve of single-stranded λDNA at room temperature (RT, 25 °C) and 37 °C (100 nm/s). As anticipated for an entropic spring, the restoration force was higher at 37°C.

(C) Loading rates ($\Delta F/\Delta t$) were calculated and presented as a function of force. The change in force per extension ($\Delta F/\Delta x$) was derived from the force-extension data in (B), which was then multiplied by the stretching speed ($\Delta x/\Delta t$; 20, 50, 100, or 300 nm/s) to obtain the rate. Linear regression lines were added for interpolation.

(D) Dehybridization force of five DNA probes at RT at four different stretching speeds (20, 50, 100, and 300 nm/s). The probe sequence information is shown in Table S1.

(E) Dehybridization force of the five probes at 37 °C and a stretching speed of 100 nm/s.

(F-G) Dehybridization force histograms of two shortened probes (15 bp, RT, 100 nm/s).

(H) Dehybridization force of a PNA oligonucleotide (RT, 100 nm/s).

Each data point was acquired from independent stretching experiments using seven or more distinct template DNA molecules. Dehybridization force was determined using Gaussian distribution fitting, represented by red lines (D-H).

(I) The difference in dehybridization time and dehybridization force between two 18 bp probes with different GC contents (39% and 61%) was examined. The data were collected when both probes bound to a template DNAs (21 independent stretching using 10 different DNA molecules). The template DNAs were stretched at a speed of 100 nm/s at 37 °C.

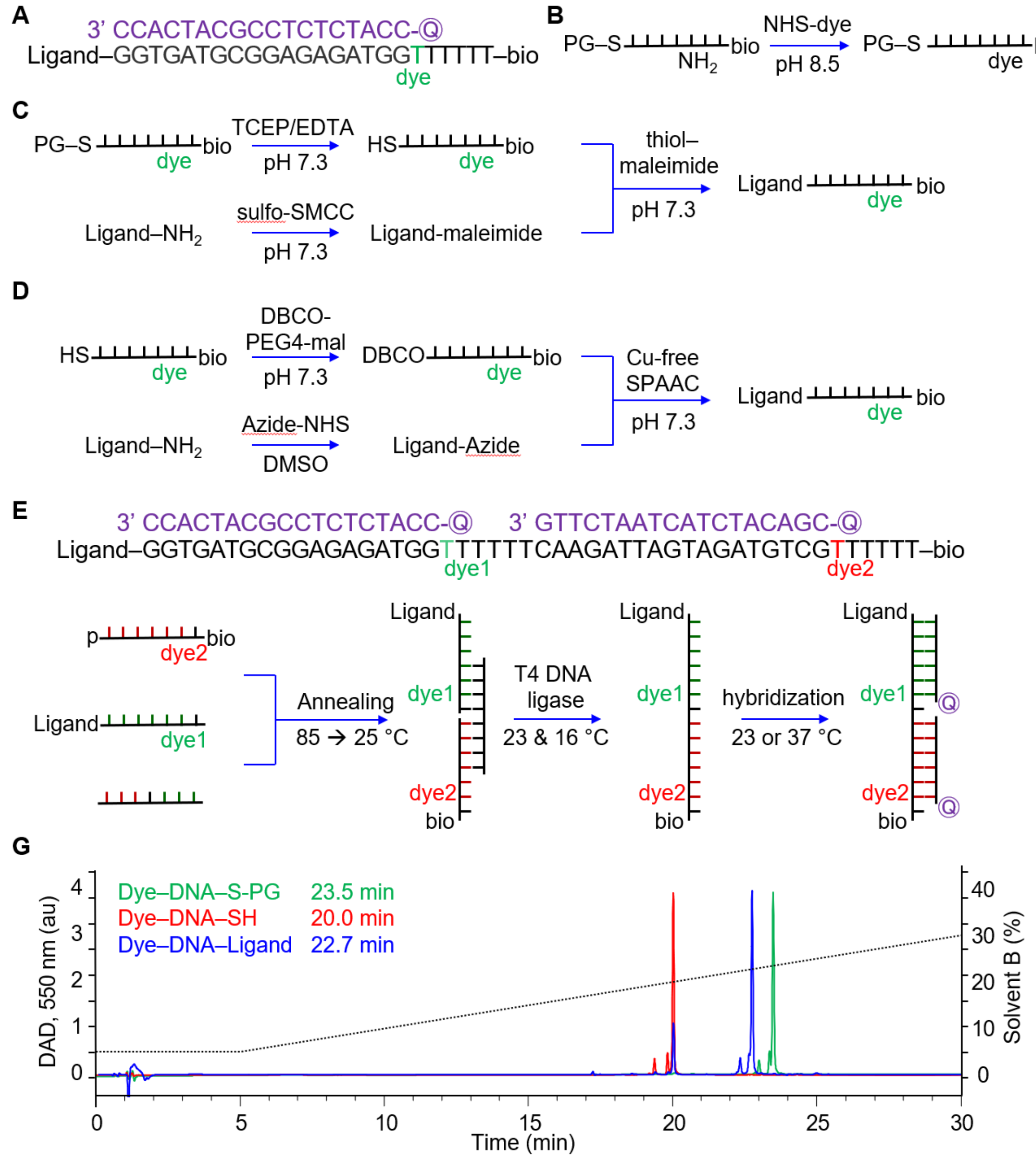

Fig. S2. OTS Synthesis Process

(A) Structure of OTS for single-level force detection. The dp30 sequence is shown. The overstretching strand contains a peptide ligand at one end, while the other end is biotinylated for surface immobilization. A quencher on the quencher strand suppresses the fluorescence signal of the dye in the overstretching strand. Ethanol precipitation was done after the reactions with DNA oligonucleotide. Each reaction was confirmed using reverse-phase High-Performance Liquid Chromatography (HPLC) and ligand conjugation products and ligation products were purified using HPLC.

(B) Conjugation of a dye or quencher to oligonucleotides using NHS ester reaction with a 20-fold molar excess of dye or quencher.

(C) Conjugation of a peptide ligand to the overstretching strand using SMCC crosslinker. The molar ratio of oligonucleotide to crosslinker to ligand was 1:11:25.

(D) Conjugation of a peptide ligand to the overstretching strand using copper-free Strain-Promoted Azide–Alkyne Cycloaddition (SPAAC). The molar ratio of oligonucleotide to crosslinker and ligand to crosslinker was 1:125 and 1:2.5, respectively. The molar ratio of oligonucleotide (DBCO) to ligand (azide) was 1:20.

(E) Synthesis of OTS for two-level force detection. The dp16/dp36 sequence is shown. Two OTS sequences were ligated. The molar ratio of three oligonucleotides was 1:1:1.

(G) Representative diode-array detection (DAD) traces of the reaction products (dp16) after dye labeling (Cy3), deprotection, and ligand conjugation (cRGDfK). HPLC purification was followed. Reverse phase HPLC was conducted using 0.05 M triethylamine acetate in water (solvent A) and acetonitrile (solvent B). The sample was injected into a C18 column at 60 °C with 5% solvent B, and the percentage of solvent B was increased by 1 %/min.

Thiol group, SH; Phosphate, p; Primary amine, NH_2; N-Hydroxysuccinimide Ester, NHS; Maleimide, mal; Biotin, bio; Protection group of thiol modification of DNA, PG-S; Quencher, Ⓠ ; Arbitrary unit, au.

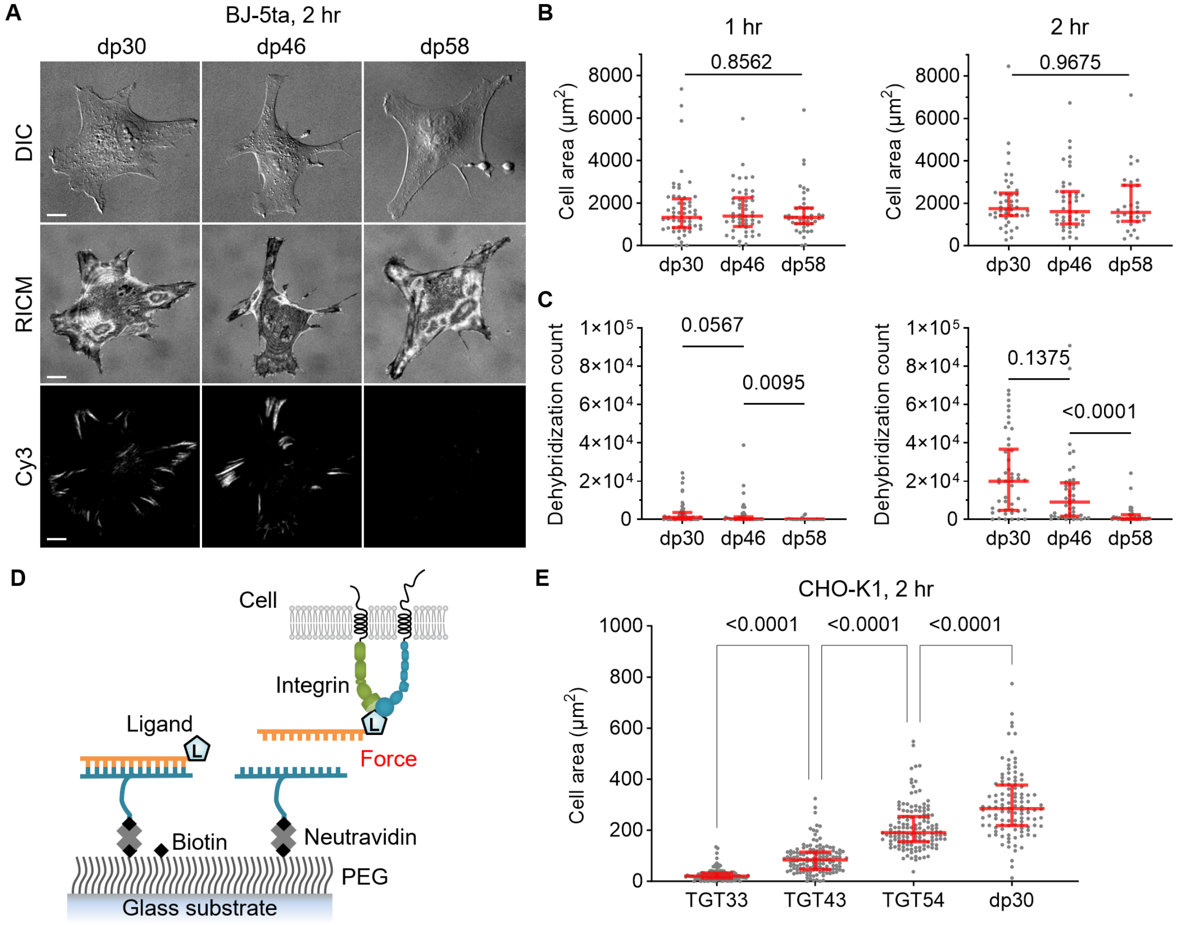

Fig. S3. Integrin αV mediated cell spreading and force transmission.

(A-C) Human foreskin fibroblasts (BJ-5ta) are seeded onto a dish coated with dp30, dp46, or dp58 (100 nM) conjugated with cRGDfK as a ligand. BJ-5ta spreading is primarily mediated by integrin αVβ1 on the cRGDfK surface.

(A) Representative cell images after 2 hours incubation. An identical contrast was applied to all three OTS (Cy3) images. Scale bars, 10 μm.

(B) Cell area on three OTSs after one (left panel) or two hours (right panel). N = 56, 49, 44; 44, 43, 32 cells from three independent experiments.

(C) The number of integrins force-activated OTS after one (left panel) or two hours (right panel). N = 59, 52, 45; 46, 44, 35 cells from three independent experiments.

(D) Schematic of tension gauge tether (TGT). TGT is designed to be ruptured by mechanical force, controlling the maximum force a single integrin can experience.

(E) Three TGTs with a tension tolerance of 33 pN, 43 pN, or 54 pN were compared with OTS. All tension probes were conjugated with cRGDfK and immobilized densely on a dish (100 nM). CHO-K1 cells were seeded for two hours. N = 106, 134, 138, 112 cells from three independent experiments.

The red lines are median and interquartile range. P-values were determined by one-way ANOVA (cell area) or Kruskal-Wallis test (dehybridization count).

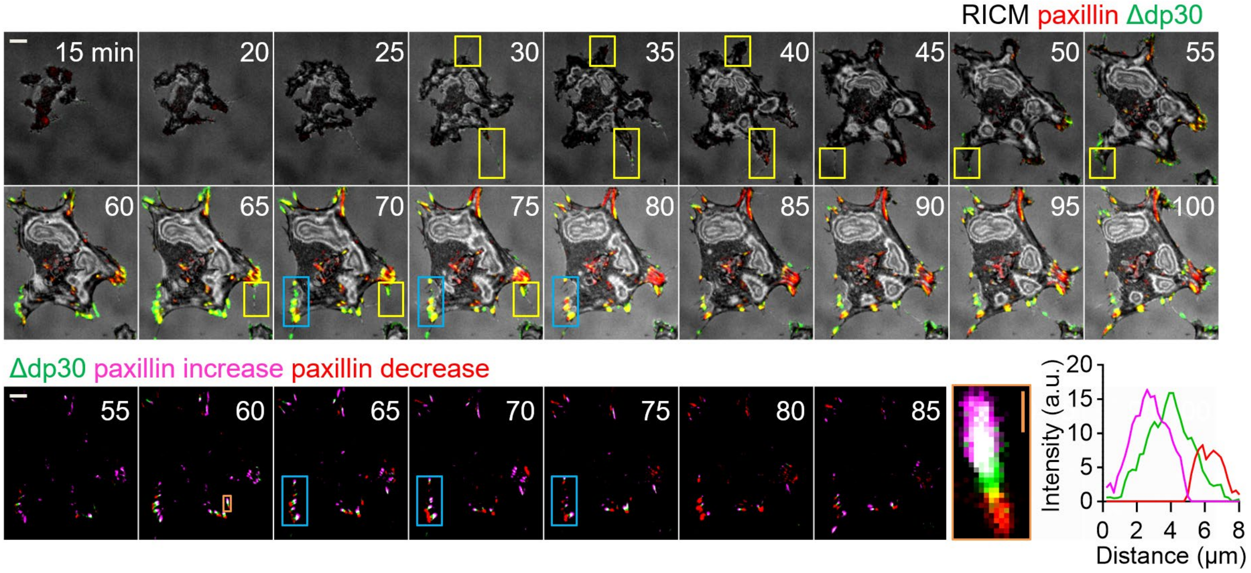

Fig. S4. Dynamic force transmission in focal adhesion.

Temporally resolved dp30 signal increase (Δdp30) reveals the integrin force (30 pN) transmission events in BJ-5ta fibroblasts with high spatial resolution and detection sensitivity. The force signals are overlaid with RICM (gray) images and paxillin-GFP (red) signals (top panel). Yellow and blue boxes indicate protruding and retracting regions, respectively. The paxillin-GFP signal increase (magenta) and decrease (red) are pseudo-colored and overlaid with the force signal (bottom panel). The intensity profiles across a focal adhesion (orange box) are shown. White scale bars, 10 µm. Orange scale bar, 2 µm.

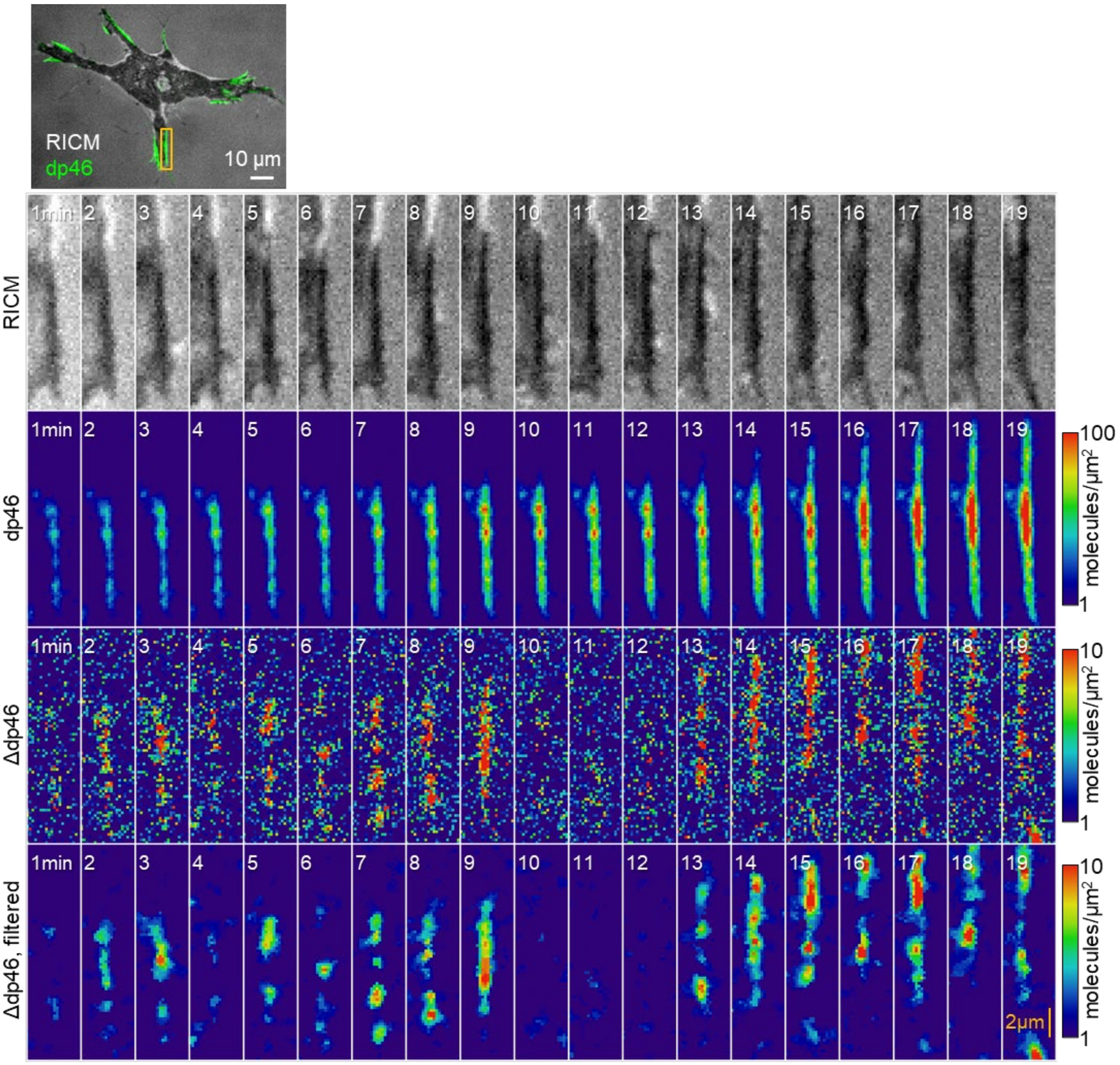

Fig. S5. Force recording after OTS refreshing.

BJ-5ta fibroblasts were seeded on a dp46 (cRGDfK, Cy3; 100 nM) coated surface. After cell spreading (~90 min), the force-activated OTS signal was removed by adding quencher strands (200 nM), and the force recording was restarted by washing out them. Time-lapse imaging was obtained at 1 min time intervals, and the fluorescent signal intensity was calibrated using single dye intensity to quantify the force transmission events. A RICM image of a cell is overlaid with the dp46 signal (top panel). The magnified RICM images (1st row) and intensity-calibrated dp46 signal (2nd row) are shown for the yellow-boxed region (bottom panel). Temporally resolved OTS signals were shown without and with applying a median filter (3rd and 4th row, ImageJ, n=2). The quantification was done without filtering.

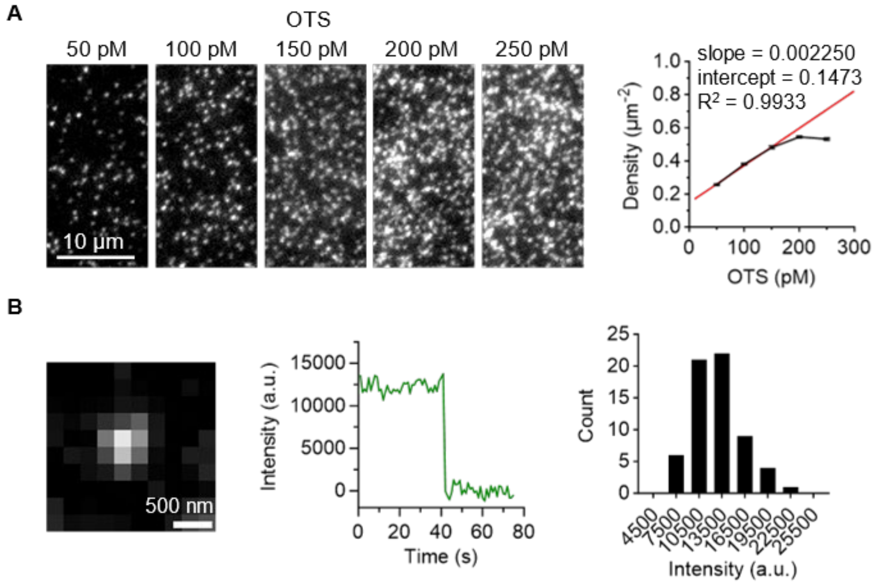

Fig. S6. OTS immobilization density measurements

Immobilization density was quantified using a counting-based method at low density and an intensity-based method at high density.

(A) At low densities, where fluorescent spots did not overlap, the number of OTS molecules was directly counted. OTS (cRGDfK-dp16/36) were incubated in microfluidic chambers (80 µm height) at five different concentrations (Atto647N images, left panel) for 5 min. Data is mean and standard deviation from five loci (4225 μm^2, 300 by 300 pixels). When the concentration exceeded 150 pM, fluorescent spots began to overlap, underestimating the density (right panel). Linear regression was performed using non-overlapping condition data (50, 100, and 150 pM), and the fitting result was used to estimate the density of slightly overlapping conditions.

(B) Fluorescence signal intensity was converted to molecular density for high-density conditions that do not allow single-molecule spot counting. Single-molecule fluorescence spots without overlapping were found (left panel) and the intensity change was monitored (middle panel) where the fluorophore density was low. The low-density regions were prepared in the periphery of the imaging dish or another chamber on the same coverglass. The intensity change due to photobleaching was collected (right panel) and the median value was used as the single fluorophore intensity.

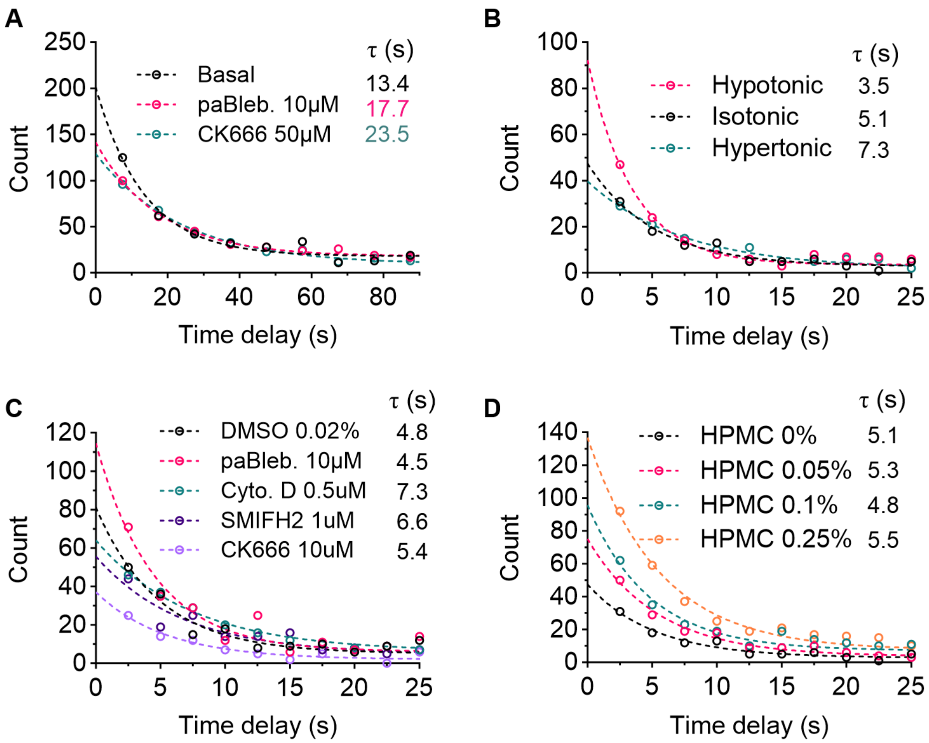

Fig. S7. Force loading rate measurements using two-level OTS

The time delay between dp16 (Atto647N) and dp30 (Cy3) signals from individual dp16/dp30 molecules was collected. Single-exponential fitting results are shown (dashed lines and τ values). To prepare the optimized OTS density for single-molecule signal monitoring, 250 pM dp16/dp30 (0.7 μm^{-2}) was incubated in the chamber, followed by incubation of cRGDfK (50 nM; 110 μm^{-2}) or LDVP (5 nM; 11 μm^{-2}) with a DNA linker with the same length.

(A) The time delay was measured for U2-OS cells seeded for about 90 min. Data was pooled from 6, 5, and 5 independent experiments (n = 870, 768, 694 colocalized spots). The field of view of each experiment (130 by 130 μm) monitored 4-7 cells.

(B-D) The time delay was measured during THP-1 cell adhesion and spreading. The field of view of each experiment (130 by 130 μm) monitored 25-35 cells.

(B) THP-1 cells were seeded in isotonic (L15 medium), hypotonic (0.5X L15), and hypertonic (L15 supplemented with 200 mM sucrose). Data was pooled from three independent experiments (n = 288, 344, 252 colocalized spots).

(C) THP-1 cells were seeded in the presence of DMSO or inhibitor after 5 min preincubation. Data was pooled from three independent experiments (n = 458, 623, 530, 434, 254 colocalized spots).

(D) THP-1 cells were seeded in the presence of HPMC. Data was pooled from three independent experiments (n = 288, 391, 618. 687 colocalized spots).

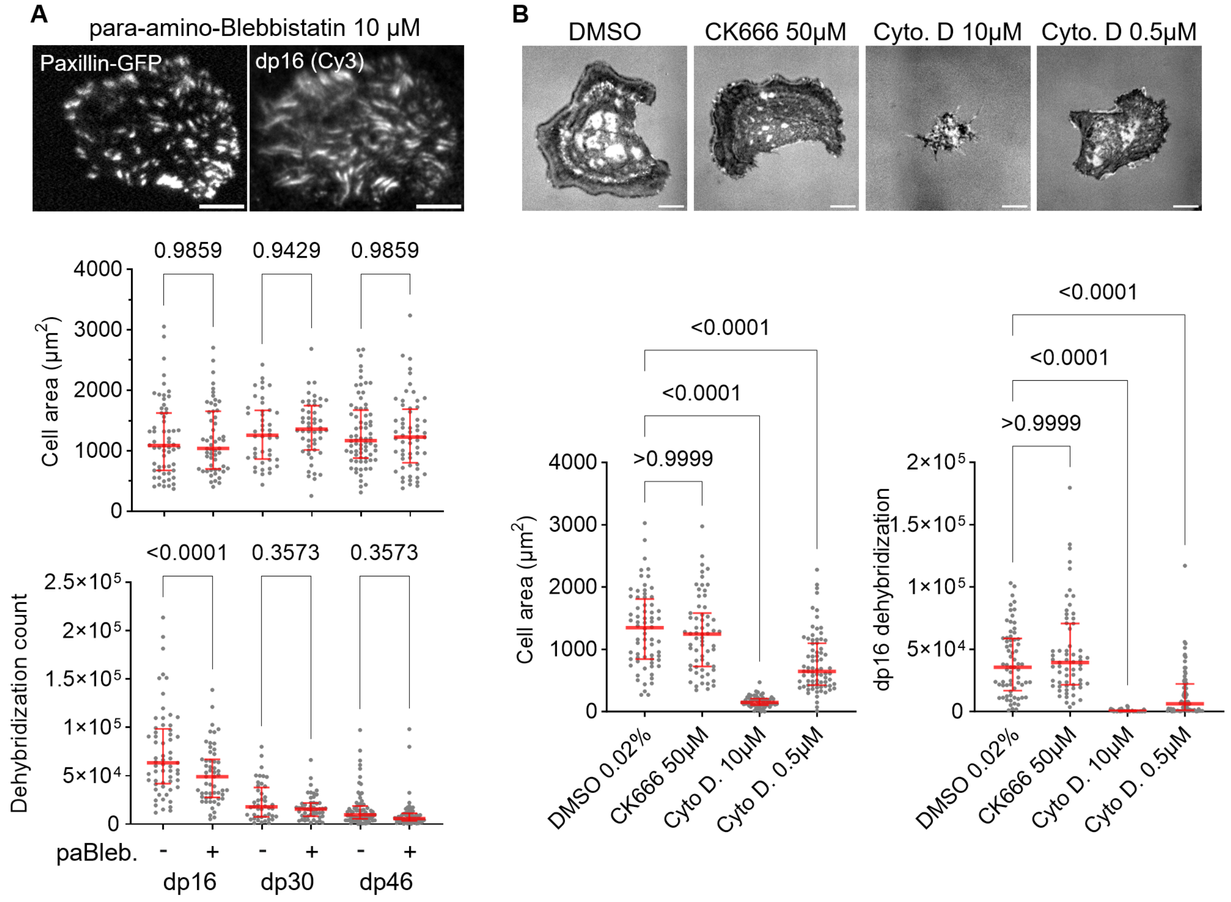

Fig. S8. Spreading and force transmission of epithelial cells

The effects of cytoskeletal inhibitors on human epithelial-like cell (U2-OS) spreading and force transmission. Cells were pre-incubated for 5 min and then seeded on dishes coated with the indicated OTS (cRGDfK/Cy3; 100 nM) for two hours at 37°C with DMSO (0.02%) or with the specified inhibitor. Cell area or the total number of dehybridized OTS was measured based on RICM image or fluorescent signal intensity, respectively. The red lines are median and interquartile range. P-values were determined by one-way ANOVA.

(A) Actomyosin activity was inhibited using para-amino-Blebbistatin (10 μM). U2-OS cells formed focal adhesions generating streak-like force transmission traces (top panel). The mean dehybridization count decreased (bottom panel; 31% for dp16, 26% for dp30, and 36% for dp46) while cell area remained constant for all conditions (middle panel; p=0.6443). Data were pooled from three independent experiments (n = 60, 55, 44, 50, 72, 60 cells).

(B) Actin polymerization was inhibited using CK666 (50 μM) and Cytochalasin D (10 or 0.5 μM). CK666 stabilizes the inactive state of Arp2/3 which generates a branched actin structure. Cytochalasin D inhibits actin polymerization by binding to G-actin. Representative RICM images

of cells spreading on dp16 surfaces are shown in the top panel and the quantification is shown in the bottom panel. The effect of CK666 on cell spreading and force transmission frequency was negligible, but Cytochalasin D (10 μM) abolished U2-OS cell spreading and force transmission. The low concentration of Cytochalasin D (0.5 μM) allowed cell spreading but the force transmission was still significantly suppressed. Data were pooled from three independent experiments (n = 68, 63, 58, 76 cells).

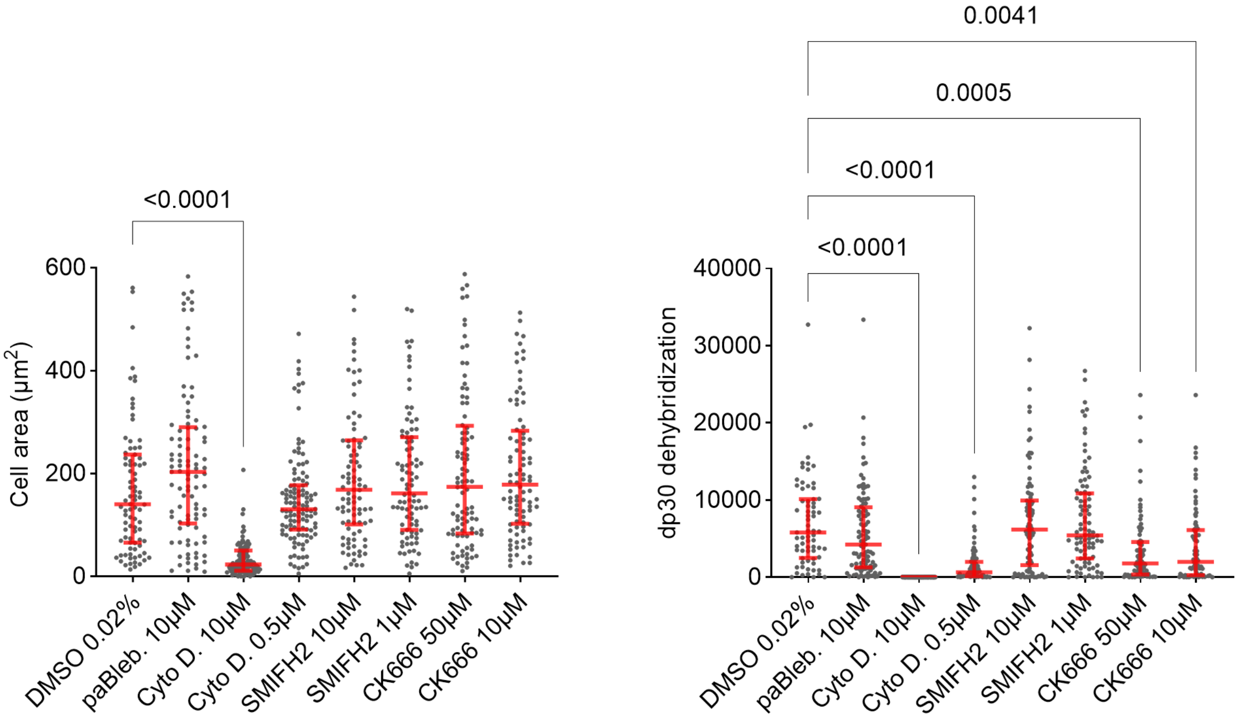

Fig. S9. Spreading and force transmission of monocytes
The effects of cytoskeletal inhibitors on THP-1 cell spreading and force transmission were examined. Cells were pre-incubated in a tube for 5 minutes and then seeded for 20 minutes at 37°C on dishes coated with LDVP-dp16/dp30 (10 nM) with DMSO (0.02%) or with the specified inhibitor. Cell area or the number of dehybridized OTS was measured based on RICM image or fluorescent signal intensity, respectively. Data were pooled from three independent experiments (n = 69, 97, 121, 116, 94, 91, 91, 92 cells). The red lines are median and interquartile range. P-values (vs. DMSO control) were determined by one-way ANOVA. All p-values not shown are >0.9999 (vs. DMSO control).

A. Probes for Dehybridization Force Measurement

OTS	Type	nt	GC%	Sequence (5' to 3')	T_m (°C)	λDNA position
dp16	DNA	18	38.9	/Cy3/CGACATCTACTAATCTTG	56.0	25830
	DNA	18	50.0	/Cy3/GTCAGCAGTATCGGTAAG	61.4	9324
dp30	DNA	18	61.1	/Cy3/CCATCTCTCCGCATCACC	67.1	37551
dp46	DNA	18	83.3	/Cy3/GCGACTGCCACGGCCCCG	78.9	32375
dp58	DNA	25	84.0	/Cy3/CGCGCCGCCAGCACGTCCGCCGGAC	85.6	20106
	DNA	15	40.0	/Cy3/CGACATCTACTAATC	50.6	25830
	DNA	15	60.0	/Cy3/CCATCTCTCCGCATC	61.1	37551
	PNA	18	83.3	/Cy3/GCGACTGCCACGGCCCCG	89.5	32375

B. Quencher strands of OTS

OTS	Type	nt	GC%	Sequence (5' to 3')	T_m (°C)	λDNA position
dp16	DNA	18	38.9	/BHQ-2/ CGACATCTACTAATCTTG	56.0	25830
dp30	DNA	18	61.1	/BHQ-2/ CCATCTCTCCGCATCACC	67.1	37551
dp46	DNA	18	83.3	/BHQ-2/ GCGACTGCCACGGCCCCG	78.9	32375
dp58	DNA	25	84.0	/BHQ-2/ CGCGCCGCCAGCACGTCCGCCGGAC	85.6	20106

C. Overstretching strands of OTS

OTS	Type	nt	Sequence (5' to 3')
dp16	DNA	24	/Ligand/CAAGATTAGTAGATGTCG/T-Cy3/TTTTT/biotin/
dp30	DNA	24	/Ligand/GGTGATGCGGAGAGATGG/T-Cy3/TTTTT/biotin/
dp46	DNA	24	/Ligand/CGGGGCCGTGGCAGTCGC/T-Cy3/TTTTT/biotin/
dp58	DNA	31	/Ligand/GTCCGGCGGACGTGCTGGCGGCGCG/T-Cy3/TTTTT/biotin/
dp16/dp30	DNA	48	/Ligand/GGTGATGCGGAGAGATGG/T-Cy3/TTTTT CAAGATTAGTAGATGTCG/T-Atto647N/TTTTT/biotin/

Table S1. Oligonucleotides for OTS

Sequence and modification information of oligonucleotides.

(A) Fluorescent probe (Cy3) conjugated DNA (5' end) and PNA oligonucleotides (N terminus) were used for dehybridization force measurements. The sequences used as OTS were specified. The melting temperature (T_m) of DNA was determined for 4 μM oligonucleotides in the presence of 150 mM Na^+ by using IDT OligoAnalyzer. The melting temperature of the PNA/DNA hybrid was predicted using the PNA Bio PNA Tool for 4 μM PNA. The sequences were borrowed from λDNA and the corresponding positions in λDNA are specified.

(B) Four characterized sequences were used as the quencher strands of OTS. A fluorescent quencher (BHQ-2) was conjugated at the 5' end.

(C) Overstretching strands of OTS include the sequence complementary to the corresponding quencher strand and modification for a fluorescent probe and ligand conjugation. T-probe indicates the dye conjugated using internal amine modification with 6C linker on thymine.

Movie S1. Force Transmission in Fibroblast Spreading
Human foreskin fibroblast (BJ-5ta) was seeded on an OTS (dp30) surface, and spreading was monitored at 5 min intervals. The movie displays RICM images for the ventral surface (left panel), OTS signals (middle panel), and calibrated force transmission event density (right panel). In the middle panel, the raw OTS signal is represented in magenta, while the increase in OTS signal is depicted in green. The calibrated density is color-coded according to the color scale shown in the right panel. Scale bar, 10 µm.

Movie S2. Two-level Force Detection in Epithelial Cells.
Human bone osteosarcoma epithelial cells (U2-OS) were seeded on a two-level OTS (dp16/dp30) surface for one hour, and force transmission was monitored at 5 s intervals after OTS refreshing. The movie displays the dp16 signals in red (Atto647N) and dp30 signals in green (Cy3), which are overlaid on RICM images (left panel) and a paxillin-GFP image (right panel). Yellow circles highlight the appearance of dp16 signals for the spots that show both dp16 and dp30 signals. Scale bar, 10 µm.

Movie S3. Two-level Force Detection in Monocytes.
Human leukemia monocytes were seeded on a two-level OTS (dp16/dp30) surface, and force transmission was monitored at 2.5 s intervals. The movie displays the dp16 signals in red (Atto647N) and the dp30 signals in green (Cy3), which are overlaid on RICM images. Yellow circles highlight the appearance of dp16 signals for the spots that show both dp16 and dp30 signals. Scale bar, 10 µm.
